# Muscle Hyperplasia in Japanese Quail by Single Amino Acid Deletion in MSTN Propeptide

**DOI:** 10.3390/ijms21041504

**Published:** 2020-02-22

**Authors:** Joonbum Lee, Dong-Hwan Kim, Kichoon Lee

**Affiliations:** 1 Department of Animal Sciences, The Ohio State University, Columbus, OH 43210, USA; lee.3920@osu.edu (J.L.); kim.4094@osu.edu (D.-H.K.); 2 The Ohio State University Interdisciplinary Human Nutrition Program, The Ohio State University, Columbus, OH 43210, USA

**Keywords:** myostatin, skeletal muscle, genome editing, quail, muscle hyperplasia

## Abstract

Mutation in myostatin (*MSTN*), a negative regulator of muscle growth in skeletal muscle, resulted in increased muscle mass in mammals and fishes. However, *MSTN* mutation in avian species has not been reported. The objective of this study was to generate *MSTN* mutation in quail and investigate the effect of *MSTN* mutation in avian muscle growth. Recently, a new targeted gene knockout approach for the avian species has been developed using an adenoviral CRISPR/Cas9 system. By injecting the recombinant adenovirus containing CRISPR/Cas9 into the quail blastoderm, potential germline chimeras were generated and offspring with three base-pair deletion in the targeted region of the *MSTN* gene was identified. This non-frameshift mutation in *MSTN* resulted in deletion of cysteine 42 in the MSTN propeptide region and homozygous mutant quail showed significantly increased body weight and muscle mass with muscle hyperplasia compared to heterozygous mutant and wild-type quail. In addition, decreased fat pad weight and increased heart weight were observed in *MSTN* mutant quail in an age- and sex-dependent manner, respectively. Taken together, these data indicate anti-myogenic function of *MSTN* in the avian species and the importance of cysteine 42 in regulating *MSTN* function.

## 1. Introduction

Poultry is one of the most important meat sources in our diet and increased meat yield can bring a huge economic benefit to the poultry industry. To enhance meat yield, genetic selection for a bigger chicken has been applied traditionally and resulted in great success with bigger body weight and higher feed efficiency [1]. Among genetic factors that contribute to muscle growth, myostatin (*MSTN*), also known as growth differentiation factor 8, is one of the most well-known and prominent genes that can be targeted to increase muscle growth. *MSTN* is mainly expressed in skeletal muscle and negatively regulates the growth of muscle [2]. This inhibitory effect on muscle growth has been further confirmed by *MSTN* mutation resulting in increased muscle mass in humans and animals including cattle, dogs, pigs, goats, sheep, rabbits, and fishes [3,4,5,6,7,8,9,10,11,12,13]. 

As a member of the transforming growth factor-β (TGF-β) superfamily, the *MSTN* gene is translated into a precursor protein (prepro-MSTN) and prepro-MSTN consists of three parts, N-terminal signal peptide, MSTN propeptide, and C-terminal mature MSTN domain [14]. The prepro-MSTN undergo three proteolytic processes to give rise to functional mature MSTN. Initially, pro-MSTN is generated by removal of signal peptide from prepro-MSTN and forms a homodimer. Subsequently, furin, a calcium-dependent serine protease, cleaves the proteolytic processing site (RXXR) between propeptide and mature MSTN [15]. After the cleavage of pro-MSTN, mature MSTN is non-covalently bound to propeptide, resulting in the formation of a latent MSTN complex [15]. For mature MSTN to be biologically active, propeptide needs to be cleaved by the bone morphogenetic protein-1/tolloid family of metalloproteinases and release mature MSTN from the latent MSTN complex [16]. The active dimer of mature MSTN binds to activin receptor type 2B (ACVR2B) and activates type-1 activin receptor serine kinases, ALK4 and ALK5. Subsequently, Smad 2 and 3 are phosphorylated and translocated to the nucleus to initiate changes in downstream gene transcription, which eventually inhibit muscle differentiation and growth [17]. 

To inhibit the anti-myogenic function of *MSTN*, the entire *MSTN* gene [10] or mature domain encoding region [7,8] can be disrupted by genetic mutations using CRISPR/Cas9, a powerful genome editing tool. On the other hand, disruption of mature MSTN binding to ACVR2B and overexpression of a dominant-negative form of ACVR2B resulted in increased muscle mass in mice [15,18]. In addition, anti-myogenic function of *MSTN* can be lost by inhibiting the proteolytic process of MSTN protein, resulting in absence of mature MSTN. In case of *MSTN* mutation in a human, insertion of 108 base-pairs in the *MSTN* gene encoding the propeptide region inhibited cleavage of pro-MSTN resulted in absence of mature MSTN and increase of muscle mass [3]. Similarly, decreased formation of mature MSTN resulted from a 12 base-pair deletion in the *MSTN* gene encoding the propeptide region in the hypermuscular mouse [19]. In avian species, alternative splicing of *MSTN* produces a truncated form of MSTN protein, MSTN-B, containing only a N-terminal half of propeptide region [20]. MSTN-B can bind to pro-MSTN and inhibit the proteolytic process of pro-MSTN [20]. Furthermore, overexpression the MSTN-B in transgenic quail increased muscle mass [21]. Accordingly, disruption of proteolytic process by mutating MSTN propeptide can be a good alternative way to down-regulate MSTN function and increase muscle mass.

The function of *MSTN* seems to be conserved in avian species as a recent study showed increased muscle mass in *MSTN* knockdown chickens [22]. To consolidate the anti-myogenic function of *MSTN* in avian species, an in vivo effect of *MSTN* mutation on avian muscle growth should be investigated. Conventionally, primordial germ cells (PGCs) have been genetically modified in vitro and injected into recipient embryos to generate a genome-edited chicken as PGCs can pass their genome to the next generation [23,24,25,26,27,28]. Recently, a new avian genome editing method using adenoviral CRISRP/Cas9 vector was reported and a first genome-edited quail was generated without PGC culture by injecting adenoviral CRISPR/Cas9 into quail blastoderm [29]. In the present study, *MSTN* was targeted in quail in vivo using the adenovirus-mediated method. Interestingly, *MSTN* mutant quail with three base-pair deletion, causing deletion of cysteine at the 42^th^ amino acid residue (C42del) in the propeptide region was generated. Quail carrying homozygous C42del mutation (*C42del/C42del*) showed significantly increased body weight and muscle mass compared to heterozygous mutant (*WT/C42del*) and wild-type (*WT/WT*) quail. Characterization of muscle growth in *C42del/C42del* quail will deepen our understanding of the role of *MSTN* in avian muscle growth. 

## 2. Results

### 2.1. Generation of MSTN Mutation in Quail Using the Adenovirus-Mediated Method

**Figure 1:**
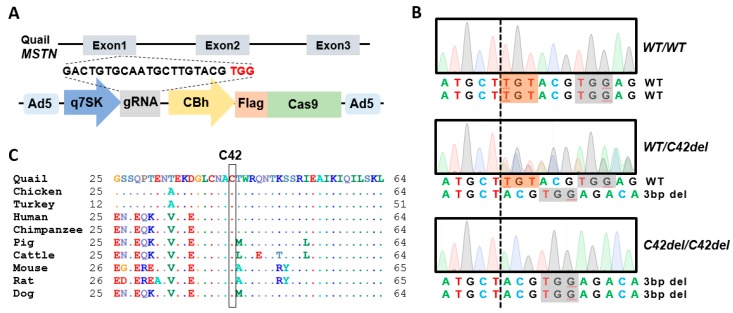
Generation of myostatin (*MSTN)* mutation in quail using adenovirus. (**A**) To target quail *MSTN*, guide RNA (gRNA) was designated on exon 1 and expression of gRNA and Cas9 was regulated by a quail 7SK promoter and a CBh promoter, respectively, in the adenoviral CRISRP/Cas9 vector. (**B**) Sanger sequencing chromatograms of a targeted region in the *MSTN* gene of wild-type (*WT/WT*), *MSTN* heterozygous mutant (*WT/C42del*), and *MSTN* homozygous mutant (*C42del/C42del*) quail were compared. Dashed line indicates the point where three base-pair deletion occurs and nucleotides that will be deleted in mutant allele are highlighted in red. Protospacer adjacent motif is highlighted in gray. (**C**) Amino acid sequences of MSTN protein after signal peptide are compared across species. Dots represent identical amino acids to quail MSTN protein. Black box indicates conserved cysteine 42 residue that is deleted by *MSTN* mutation in quail.

Quail *MSTN* gene has three exons and two introns, and guide RNA (gRNA) was designed to target exon 1 (Figure 1A). Expression of gRNA and Cas9 expression was regulated by a quail 7SK promoter and a CBh promoter, respectively (Figure 1A), and confirmed in our previous in vitro and in vivo studies [29,30]. After production of recombinant adenovirus type 5 containing *MSTN* gRNA and Cas9 expression cassettes, the adenovirus was injected into the quail blastoderm to generate potential germline chimeras. Offspring were produced from mating of potential germline chimeras with wild-type quail and three base-pair deletion in the targeted region of *MSTN* gene was identified from offspring genotypes. Heterozygous mutant offspring were further mated to generate *WT/WT*, *WT/C42del*, and *C42del/C42del* quail (Figure 1B). *MSTN* gene with non-frameshift three base-pair deletion mutation encoded MSTN protein without cysteine 42 residue, strictly conserved across species (Figure 1C).

To analyze off-target mutations in C42del mutant offspring, six potential off-target regions were selected based on high homologous scores and the existence of protospacer adjacent motif (PAM) sequences using the NCBI Genome BLAST. The potential off-target regions shared 12 to 15 matched nucleotides of the 20 nucleotides gRNA sequences (Table A1). The potential off-target regions were amplified by polymerase chain reaction (PCR) using genomic DNA from heterozygous mutant offspring, and off-target mutation was not identified by sequence analysis of the PCR products.

### 2.2. Positive Effect of MSTN C42del Mutaiton in Quail Muscle Growth

**Figure 2:**
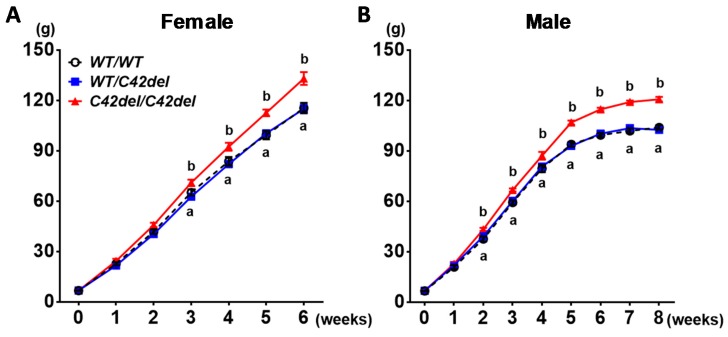
Comparison of body weights among groups in female (**A**) and male (**B**) quail. Body weights were measured weekly from day of hatching (0) to 6 weeks in female and 8 weeks in male. One-way ANOVA followed by Tukey’s multiple comparisons test was used for statistical analysis by the Graphpad PRISM 6.02 program. Values present means ± standard error of the mean (SEM). *n* = 11 in female and 10 in male. ^a^ Means include both *WT/WT* and *WT/C42del* quail at each time point in each sex. ^a–b^ Means sharing the same superscript at each time point are not significantly different from each other in each sex (*p* < 0.05).

After generation of *WT/WT*, *WT/C42del*, and *C42del/C42del* quail, body weights were measured weekly until 6 weeks in the female and 8 weeks in the male (Figure 2). Female quail were euthanized for tissue sampling at week 6 before egg laying, because laying of approximately 8.7 grams of egg causes a fluctuation of body weight, and fat mobilization from body fat to support egg yolk production affects fat pad weights [31]. Male quail were euthanized at week 8 for tissue sampling after body weight reached a plateau. As shown in Figure 2, female and male *C42del/C42del* quail showed significantly increased body weight from week 3 and week 2 compared to *WT/C42del* and *WT/WT* quail, respectively. However, there was no difference in body weight between *WT/C42del* and *WT/WT* quail. Average of final body weight from *C42del/C42del* quail was approximately 20% heavier in females and 30% heavier in males compared to other groups. 

**Figure 3:**
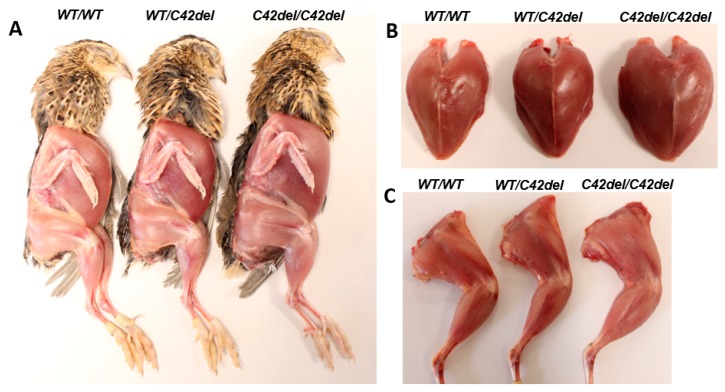
Phenotypic comparisons of whole body (**A**), breast muscle (**B**), and leg muscle (**C**) among groups in 6-week old female quail.

As shown in Figure 3, *C42del/C42del* quail showed wide and bulged breast and a thicker leg compared to those of other groups. To assess the effects of C42del mutation on muscle growth, pectoralis major and minor from the breast, biceps femoris, semitendinosus, gastrocnemius from the leg, and tricep brachii from the wing were weighed [2,32]. All these muscles from *C42del/C42del* quail were significantly heavier compared to other groups in females (Table 1) and males (Table 2). In case of fat pad weight, female *C42del/C42del* quail showed a significantly lower weight of leg and abdominal fat pads compared to other groups (Table 1). However, there was no significant difference in leg and abdominal fat pad weights among three groups in 8- and 12-week old male quail, although a trend of low leg and abdominal fat pad weights were observed in *WT/C42del* and *C42del/C42del* quail compared to *WT/WT* quail (Table 2). Contrary to the fat pad weights, heart weight was significantly heavier only in male *WT/C42del* and *C42del/C42del* quail compared to *WT/WT* quail (Table 2), whereas female quail showed similar heart weight among three groups (Table 1). These results indicate that C42del mutation affects not only skeletal muscle weights, but also the weights of adipose tissue and heart.

### 2.3. Muscle Fiber Hyperplasia in C42del/C42del Quail

**Figure 4:**
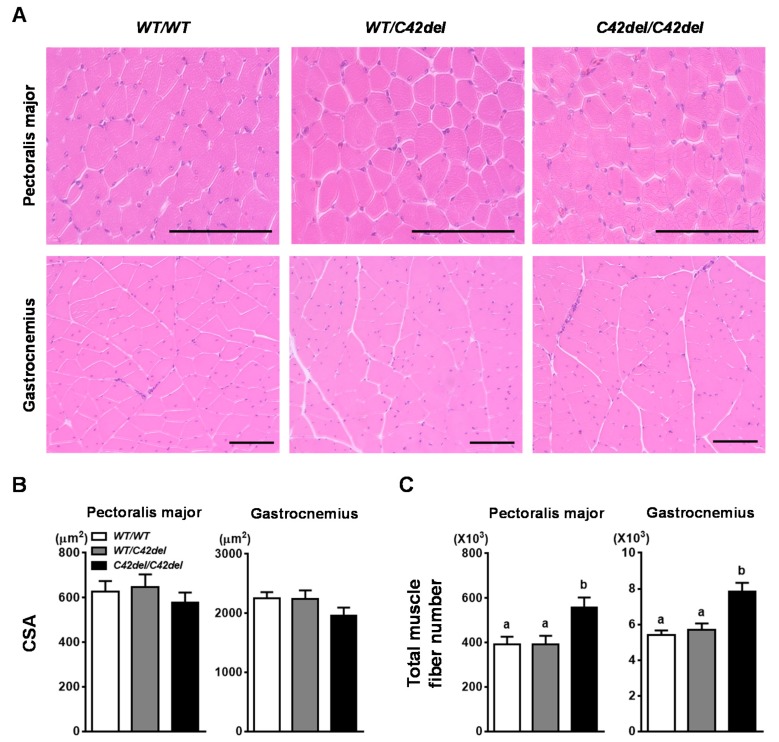
Morphological differences of pectoralis major and gastrocnemius muscles among groups in 6-week old female quail. (**A**) Histological comparison of hematoxylin and eosin stained pectoralis major and gastrocnemius muscles. Scale bar: 100 μm. (**B**) Comparison of muscle fiber cross-sectional area (CSA). (**C**) Comparison of total muscle fiber number. The values are means ± SEM. *n* ≥ 5. Statistical analyses were performed by one-way ANOVA followed by Tukey’s multiple comparisons test using the Graphpad PRISM 6.02 program. ^a–b^ Means that have no superscript in common in a graph are significantly different (*p* < 0.05).

To compare the histological difference between *C42del/C42del* and *WT/WT* quail, muscle fiber cross-sectional area (CSA) and total fiber number of pectoralis major (PM) and gastrocnemius muscles was measured as a representative muscle of breast and leg, respectively [21,33]. Average CSA of muscle fiber in both muscles among groups was not significantly different (Figure 4A,B). However, total fiber number of both muscles was significantly increased in *C42del/C42del* quail (Figure 4C). There was a 40% increase in total fiber number in PM and gastrocnemius muscle of *C42del/C42del* quail. These data showed that C42del mutation caused muscle fiber hyperplasia, rather than fiber hypertrophy in skeletal muscle, resulting in increased body weight and muscle mass of *C42del/C42del* quail.

## 3. Discussion

Previously, CRISPR/Cas9-mediated genome editing in the avian species has been conducted using PGC-mediated method and a few genome-edited chickens have been reported [24,25,26,27,28]. Without mediating PGCs, *MLPH* knockout quail was generated recently by injecting adenovirus containing CRISRP/Cas9 system into the quail blastoderm [29]. In the present study, the same adenovirus-mediated method was used and three base-pair deletion mutation in the *MSTN* gene was generated (Figure 1B), causing C42del in MSTN propeptide without off-target mutation in six potential off-target regions. Unlike one or two base-pair deletion causing frameshift mutation and complete disruption of targeted gene, three base-pair deletion in *MSTN* gene encodes intact mature MSTN, a biologically active domain. However, *C42del/C42del* quail showed significantly increased body weight and muscle mass compared to other groups, indicating compromised anti-myogenic function of MSTN protein by C42del mutation in MSTN propeptide. When *MSTN* function was inhibited by genetic mutation, propeptide, or siRNA, increased expression of myogenic factors was observed in vivo and in vitro [34,35,36,37,38,39,40]. Furthermore, the transgenic quail overexpressing MSTN-B showed increase in muscle mass with prolonged and increased expression of Pax7 in embryonic muscle [21]. Taken together, increased muscle mass by inhibition of *MSTN* might be accompanied by an increase in myogenic factors. 

Function of *MSTN* in negative regulation of muscle growth is conserved across species and natural or genetically engineered disruption of the *MSTN* gene gives increased muscle mass in mice, cattle, dogs, pigs, goats, sheep, rabbits, fishes, and humans [2,3,4,5,6,7,8,9,10,11,12,13]. However, the mechanism of enhancing muscle mass by *MSTN* mutation is somewhat different across species. In *MSTN* mutant mice, goats, and rabbits, increased muscle mass resulted from increases in muscle fiber number (hyperplasia) and size (hypertrophy) [2,8,10]. However, only muscle fiber hyperplasia was observed in *MSTN* mutant cattle and pigs [4,7]. In *C42del/C42del* quail, increased muscle mass was caused by muscle fiber hyperplasia, rather than fiber hypertrophy. In addition, muscle fiber hyperplasia was also observed in *MSTN* knockdown chickens [22]. Although both *C42del/C42del* quail and *MSTN* knockdown chickens are not complete *MSTN* knockout avian models, the data suggests that disruption of the *MSTN* gene in the avian species will increase muscle mass with muscle fiber hyperplasia, rather than fiber hypertrophy or both.

In most terrestrial vertebrates, muscle fiber number is known to be fixed before birth [41,42] and expression of *MSTN* is observed during embryonic stage when muscle fiber is developed [2,43]. Thus, *MSTN* knockout can affect muscle development from the embryonic stage and increased fetal and birth weight of *MSTN* mutant animals was observed in sheep, cattle, and goats [9,34,44]. In mammals, nutrients are continuously supplied through maternal circulation during embryonic development, which can further support enhanced muscle growth by *MSTN* mutation in fetus. However, *C42del/C42del* quail did not show increased body weight at hatching compared to *WT/C42del* and *WT/WT* quail. This suggests that fixed nutrient from the confined egg environment might not fully support the genetic predisposition of *C42del/C42del* quail toward enhanced muscle growth during the embryonic stage. Similar to our *MSTN* mutant model, body weight at hatching between wild-type and *MSTN* mutant was not different in medaka and zebrafish [11,12]. With no difference in hatching weight, the significantly increased body weight from 3 weeks in female and 2 weeks in male *C42del/C42del* quail compared to other groups suggests a genetic effect of *MSTN* mutation occurs in the post-hatch period. Interestingly, C42del mutation had haploinsufficiency in terms of increased body weight and muscle mass in the male and female, showing similar body weight and muscle mass between *WT/C42del* and *WT/WT* quail. This haploinsufficiency of *MSTN* mutation in body weight was also observed in zebrafish and catfish [12,13], which might be partially due to a unique mechanism of nutrient supply to the fetus in fishes and quail.

In addition to negative regulation of skeletal muscle growth, pleotropic effect of the *MSTN* gene on adipose tissue and the heart should be considered because *MSTN* is expressed in adipose tissue and heart, as well [2,45]. *MSTN* mutation resulted in decreased fat mass in mice [46,47], cattle [48,49], and pigs [50]. Likewise, *C42del/C42del* quail showed significantly decreased leg and abdominal fat pad weights compared to *WT/WT* quail in females, and intermediate fat pad weights were observed in *WT/C42del* quail in females. The trend of decreased fat mass in *MSTN* mutant quail was also shown in 8- and 12-week old males, but the difference was not significant. In female quail, fat deposition is significantly increased prior to the egg laying stage to reserve more fat in adipose tissue, which will be mobilized to support egg yolk synthesis [31]. However, increasing fat mass before onset of the egg laying period, found in female *WT/WT* quail at 6-week old, will be inhibited by C42del mutation, resulting in significantly lower weights of fat pads in female *C42del/C42del* quail. Contrary to female quail, fat accumulation will be gradually increased in male quail. During the growth period, growth of skeletal muscle is mostly achieved and largely contributes to the increase of body weight. To support growth at this period, more nutrients are allocated toward actively growing tissue such as skeletal muscle and fat deposition is generally increased after body weight reaches a plateau in male quail, as shown in the current study. However, no significant difference in fat pad weights among male groups at each age suggests a different response to *MSTN* mutation in fat accretion between the female and male. 

In terms of *MSTN* effect on the heart, increased heart weight was shown in *MSTN* mutant mice and rabbits [10,51]. The same trend of significant increase in heart weight by *MSTN* mutation was also observed in *WT/C42del* and *C42del/C42del* quail compared to *WT/WT* quail in males. However, there was no difference in heart weight among female groups, indicating sexual dimorphism. In various studies with genetically modified mouse models, male transgenic mice were more sensitive to genetic factors in development of cardiac diseases [52]. Additionally, *MSTN* knockout female mice showed better cardiac performances than male [51]. Similarly, when cardiac performances were investigated using meat-type heavy turkeys, a slight decrease of cardiac output value related to body surface was observed in male turkeys [53]. Considering these findings, sexual dimorphic phenotype of heart weight in *C42del/C42del* quail could be due to more sensitive to *MSTN* mutation in male. There was no sudden death in male *C42del/C42del* quail and no difference in mortality rate among groups, suggesting no major heart issues. However, it will be interesting to investigate whether increased heart weights in male *C42del/C42del* quail can be resulted from increased size of heart and/or thickening of heart muscle, which will provide basis for direction of further investigation on cardiac performances and diseases.

In the present study, C42del mutation in MSTN protein resulted in increased body weight, muscle mass, and heart weight, along with decreased fat pad weights, comparable phenotypic characteristics of other *MSTN* knockout animals. The current finding that the C42del mutation positively affected muscle growth in quail indicates an important role of the conserved cysteine 42 residue in MSTN function. Several studies demonstrated that non-frameshift mutations in MSTN propeptide inhibited proteolytic process of pro-MSTN, resulted in an absence or decrease of mature MSTN, thereby increasing muscle mass in humans and mice [3,19]. Although our trials to detect MSTN protein with commercial antibodies had been failed, it will be interesting to investigate whether proteolytic process of MSTN protein can be affected by C42del mutation in propeptide. Our study provides an important avian model with a novel mutation in MSTN propeptide for hyperplastic muscle growth and for application to improve genetic traits of poultry with increased meat yield. 

## 4. Materials and Methods

### 4.1. Animal Care

All animal care protocol and procedures were approved by the Institutional Animal Care and Use Committee at The Ohio State University (Protocol 2015A00000013-R1, Approval date: 6 March 2018). Quail were fed ad libitum and maintained at The Ohio State University Poultry Facilities in Columbus, OH, USA. Experimental male birds were euthanized at 8 and 12 weeks of ages, and female birds were euthanized at 6 weeks of age via CO^2^ inhalation. 

### 4.2. Construction and Injection of Adenoviral Vector

To target *MSTN* gene in Japanese quail, 20 nucleotide sequences of gRNA, followed by PAM sequences, 5’-NGG-3’, were designed in exon 1 based on high on-target scores and low off-target frequencies using a CRISRP gRNA design tool (benchling.com). The gRNA was inserted into our previously optimized CRISPR/Cas9 vector and the CRISPR/Cas9 expression cassettes were transferred to the adenoviral shuttle vector [29,30]. Subsequently, adenovirus containing the CRISPR/Cas9 system was commercially produced with final titer of 3.0 × 10^10^ PFU/mL. The recombinant adenovirus was injected into the EGK stage XI quail blastoderm according to our previous study [29]. Briefly, wild-type Japanese quail eggs were positioned upside down and laterally for 2 h, respectively, to position quail blastoderm on the lateral apex of the eggs. After making a small window on the lateral apex of the eggs, 2 µL of the virus (6.0 × 10^7^ PFU) was injected into the middle of the quail blastoderm using a microneedle and microinjector (Tritech Research, Los Angeles, CA, USA). The window was sealed with parafilm and the injected eggs were incubated at a temperature of 37.5 °C and 60% relative humidity until hatching occurred. 

### 4.3. Production of Quail with MSTN Mutation

After injection of recombinant adenovirus into quail eggs, potential germline chimeras were hatched and mated with wild-type quail. Eggs from chimeras were collected daily and incubated weekly. Hatched offspring were tagged and maintained in brooder cages until genotyping after 3 weeks of post-hatch. For genotyping, feather pulp was collected from each offspring to extract genomic DNA based on our previous genomic DNA extraction method using feather pulp [21,54]. In brief, 300 μL of cell lysis solution (CLS; 200 mM NaCl, 50 mM Tris-Cl, 10 mM Ethylenediaminetetraacetic acid, 1% Sodium dodecyl sulfate, pH 8.0) containing proteinase K (1.5 μL/300 μL CLS, Invitrogen, Waltham, MA, USA) was added to the feather pulp and the tube was placed in a 55 °C heat block for 4 h. After incubation, 300 μL of phenol–chloroform–isoamyl (Sigma-Aldrich, St. Louis, MO, USA) was added to the tube to remove the protein and vortexed vigorously. The mixture was centrifuged at 13,000× *g* for 5 min and the 240 μL of supernatant was transferred to a new tube. Subsequently, 80 μL of 7.5 M ammonium acetate and 240 μL of isopropanol was added, mixed vigorously, and centrifuged at 13,000× *g* for 5 min. After discarding the supernatant, the pellet was washed with 70% ethanol followed by drying. The dried pellet was dissolved in TE buffer containing RNase A (10 mg/mL, Qiagen, Valencia, CA, USA). The genomic DNA was used for PCR to amplify the targeted region in the *MSTN* gene with a primer set listed in Appendix A Table A2. Conditions for PCR were 95 °C for 90 s, followed by 40 cycles of 95 °C for 40 s, 53 °C for 40 s, 68 °C for 30 s, and a final extension of 68 °C for 5 min. The PCR bands were extracted using a QIAquick Gel Extraction Kit (Qiagen), according to the manufacturer’s protocol, and the products were sequenced at The Ohio State University Comprehensive Cancer Center. After identification of offspring carrying *MSTN* mutation in the targeted region, male and female heterozygous *MSTN* mutant offspring were mated to generate homozygous *MSTN* mutant progenies. 

### 4.4. Analysis of Off-Target Mutation

*MSTN* gRNA with PAM motif, 5’-NGG-3’, was screened against Japanese quail genome using BLAST Genome search in the PubMed Genome database (https://www.ncbi.nlm.nih.gov/genome) and six potential off-target sites with high homology scores were selected (Table A1). PCR was performed to amplify six potential off-target sites using primer sets listed in Appendix A Table A2 at the same conditions described above. The PCR bands were extracted and sequenced to detect any off-target mutations. 

### 4.5. Tissue Sampling

*WT/WT*, *WT/C42del*, and *C42del/C42del* quail were produced from *WT/C42del* parents and maintained together in the same brooder cages. Body weights were measured weekly and female and male quail were euthanized at 6 weeks and 8 weeks, respectively, using CO2 inhalation to collect muscle, adipose tissue, and heart samples. Additionally, adipose tissues were collected from 12-week old male quail. PM and pectoralis minor muscles, biceps femoris, semitendinosus, gastrocnemius, tricep brachii, and heart were collected and weighed. In addition, leg fat and abdominal fat at the lower tip of breast muscle were collected and weighed [54]. A portion of PM and right gastrocnemius were fixed in 10% neutral buffered formalin for histological analysis. 

### 4.6. Histological Processing and Measurement of Muscle Fiber Number and Size

To measure the total area of PM, whole breast muscle attached to the bone was taken and cross-sectioned as described in previous studies [55]. In short, breast muscle was cross-sectioned perpendicular to the direction of the muscle fiber and visualized using a camera (EOS Rebel T7, Canon, Japan) to calculate the whole area of PM by NIH image J software (ImageJ, Ver. 1.52, http://imagej.nih.gov/ij). Fixed PM and gastrocnemius were sectioned into 5 μm slices after embedding in paraffin. The sections were stained with hematoxylin and eosin by following general procedures described in our previous study [21] and the morphology was analyzed. The whole cross-sectional area of stained gastrocnemius was visualized under a microscope (Olympus Optical, Tokyo, Japan) using a camera (EOS Rebel T7) and measured using ImageJ. Subsequently, five regions from each slide of stained muscle samples were randomly selected using a microscope (EVOS FL imaging systems, Thermofisher Scientific, Waltham, MA, USA). The area of each region and number of muscle fiber in the region was measured using ImageJ and at least 500 fibers were analyzed from each muscle to calculate average muscle fiber CSA. Subsequently, the total area of PM and gastrocnemius was divided by CSA to calculate approximate total fiber number of each muscle sample. 

### 4.7. Statistical Analyses

All data were expressed as means ± SEM. The data were analyzed using Graphpad Prism software, version 6.02. For all comparisons in this study, multiple means were compared by one-way ANOVA followed by Tukey’s multiple comparison test. *p*-value, *p* < 0.05. was considered statistically significant.

## Figures and Tables

**Table 1:** Comparison of muscle, adipose tissue, and heart weights in 6-week old female quail.

Tissue	*WT/WT*	*WT/C42del*	*C42del/C42del*
Pectoralis Major (g)	16.19 ± 0.26 ^a^	16.15 ± 0.500 ^a^	20.12 ± 0.71 ^b^
Pectoralis Minor (g)	5.56 ± 0.10 ^a^	5.61 ± 0.16 ^a^	6.92 ± 0.20 ^b^
Biceps Femoris (g)	2.14 ± 0.06 ^a^	2.20 ± 0.04 ^a^	2.68 ± 0.08 ^b^
Semitendinosus (g)	0.95 ± 0.02 ^a^	0.98 ± 0.03 ^a^	1.17 ± 0.04 ^b^
Gastrocnemius (g)	0.80 ± 0.03 ^a^	0.79 ± 0.02 ^a^	0.10 ± 0.04 ^b^
Tricep Brachii (g)	0.56 ± 0.02 ^a^	0.54 ± 0.03 ^a^	0.69 ± 0.02 ^b^
Leg Fat (g)	0.34 ± 0.03 ^a^	0.25 ± 0.02 ^ab^	0.24 ± 0.02 ^b^
Abdominal Fat (g)	0.23 ± 0.04 ^a^	0.19 ± 0.02 ^ab^	0.16 ± 0.02 ^b^
Heart (g)	0.87 ± 0.03 ^NS^	0.87 ± 0.03 ^NS^	0.87 ± 0.02 ^NS^

The values are means ± SEM. *n* = 11. Statistical analyses were performed by one-way ANOVA followed by Tukey’s multiple comparisons test using the Graphpad PRISM 6.02 program. g: gram. ^a–b^ Means sharing the same superscript in a row are not significantly different from each other (*p* < 0.05) and ^NS^ means no significant difference.

**Table 2:** Comparison of muscle, adipose tissue, and heart weights in 8- and 12-week old male quail.

Tissue	*WT/WT*	*WT/C42del*	*C42del/C42del*
Pectoralis Major (g)	14.00 ± 0.52 ^a^	13.95 ± 0.32 ^a^	17.96 ± 0.26 ^b^
Pectoralis Minor (g)	4.93 ± 0.17 ^a^	4.59 ± 0.32 ^a^	6.35 ± 0.09 ^b^
Biceps Femoris (g)	2.02 ± 0.06 ^a^	2.06 ± 0.05 ^a^	2.58 ± 0.05 ^b^
Semitendinosus (g)	0.89 ± 0.03 ^a^	0.92 ± 0.02 ^a^	1.14 ± 0.03 ^b^
Gastrocnemius (g)	0.67 ± 0.02 ^a^	0.70 ± 0.02 ^a^	0.91 ± 0.03 ^b^
Tricep Brachii (g)	0.48 ± 0.01 ^a^	0.50 ± 0.01 ^a^	0.61 ± 0.02 ^b^
Heart (g)	0.82 ± 0.03 ^a^	0.92 ± 0.03 ^b^	0.95 ± 0.04 ^b^
Leg Fat (g)	0.41 ± 0.06 ^NS^	0.30 ± 0.032 ^NS^	0.32 ± 0.04 ^NS^
Abdominal Fat (g)	0.26 ± 0.04 ^NS^	0.17 ± 0.02 ^NS^	0.21 ± 0.03 ^NS^
Leg Fat (g, 12 weeks)	0.70 ± 0.13 ^NS^	0.65 ± 0.05 ^NS^	0.58 ± 0.09 ^NS^
Abdominal Fat (g, 12 weeks)	0.44 ± 0.08 ^NS^	0.39 ± 0.03 ^NS^	0.37 ± 0.07 ^NS^

The values are means ± SEM. Tissues without specific age are from 8-week old quail. *n* = 10 at 8 weeks of age and *n* = 8 at 12 weeks of age. Statistical analyses were performed by one-way ANOVA followed by Tukey’s multiple comparisons test using the Graphpad PRISM 6.02 program. g: gram. ^a–b^ Means sharing the same superscript in a row are not significantly different from each other (*p* < 0.05) and ^NS^ means no significant difference.

## Abbreviations

ACVR2B	activin receptor type 2B
C42del	cysteine 42 deletion
CSA	cross-sectional area
MSTN	myostatin
gRNA	guide RNA
PAM	protospacer adjacent motif
PCR	polymerase chain reaction
PGC	primordial germ cell
PM	pectoralis major
SEM	standard error of the mean
TGF-β	transforming growth factor-β
WT	wild-type

## Appendix A

**Table A1:** Potential off-target sites of *MSTN* gene in Japanese quail genome. Identical nucleotides in potential off-target sites are underlined.

Quail	Chromosome	Locus	Score	Sequence	PAM	Direction
*MSTN*	7	4,829,332	46.1	GACTGTGCAATGCTTGTACG	TGG	+
Off-target 1	1	125,916,261	30.2	TCCTAAGAAATGCTTGTACG	TGG	+
Off-target 2	7	7,649,801	28.2	AATGCTGCAATGCTTGGACG	TGG	+
Off-target 3	5	424,027	28.2	GTCTCTTAGATGCTTGTACG	TGG	−
Off-target 4	1	105,890,272	28.2	AATGGACACATGCTTGTACG	GGG	+
Off-target 5	4	22,457,528	28.2	AGTCACTCTATGCTTGTACG	GGG	+
Off-target 6	1	55,771,184	28.2	TGGTATGCAATGCCTGTACG	CGG	−

**Table A2:** List of primers used in the present study.

Purpose	Forward (5′-3′)	Reverse (5′-3′)
*MSTN*	GCATGGACGAGCTGTACAAGTA	CCCTGCTAATGTTAGGTGCTT
Off-target 1	CGCACTATGGAATGGCAAGATTT	TCTCCCTCAATCTTAGTACTGCTT
Off-target 2	AGACCTTCTGCATACTGCCTT	CTTCAGAACTTGCAGGTTTGCTA
Off-target 3	TGTGTTCAACTGCTCAGAAGGAA	GTGGGAAGTTCCAGACAAGTT
Off-target 4	ATGGGAAGAACTGCTACTGGAA	AAGAGGCTTCCTGTGCTTCT
Off-target 5	CACTGAGGAAGTTTGTCTTGGAGTTA	TGGCTGAAAGATCTTATCTTCACTCA
Off-target 6	CTGTCTCTGTGTCCAGATCAGAT	AGAGGAGCCTCATGTTGGAA
